# Henry Ingersoll Bowditch: The Prominent Physician Who Introduced Thorax Examination and Trocar Thoracentesis in the United States of America

**DOI:** 10.7759/cureus.52582

**Published:** 2024-01-19

**Authors:** Theoni Zougou, Nikolaos Pappas, Evaggelos Mavrommatis

**Affiliations:** 1 Department of Anatomy, National and Kapodistrian University of Athens School of Medicine, Athens, GRC

**Keywords:** public health, american medicine, valvular heart disease, paracentesis thoracis, stethoscope

## Abstract

Born in a wealthy family with a tradition in science, Henry Ingersoll Bowditch (1808-1882) with studies at Harvard Medical School and in Europe had succeeded in leaving his mark in the American history of medicine. He had been a pioneer in the stethoscope’s use, which was promoted and suggested to all physicians of his era. He had widely used thoracentesis, an ancient procedure, for pleuritic effusions, diagnosed with a stethoscope. Inside his most popular treatise “The Young Stethoscopist,” he had given a plethora of data concerning the auscultation of the lungs, heart, and vessels; obstetrics; and veterinary. To help younger physicians, he demonstrated through figures local anatomy and positions for auscultation, providing information for various types of stethoscopes being in use during the mid-19th century. He was a humanist and reformer for public hygiene. This historical vignette aims to present Henry Ingersoll Bowditch and his work concerning the thorax. For his contributions to education and public hygiene, he should be celebrated as one of the most important figures of the eve of American medicine.

## Introduction and background

**Figure 1:**
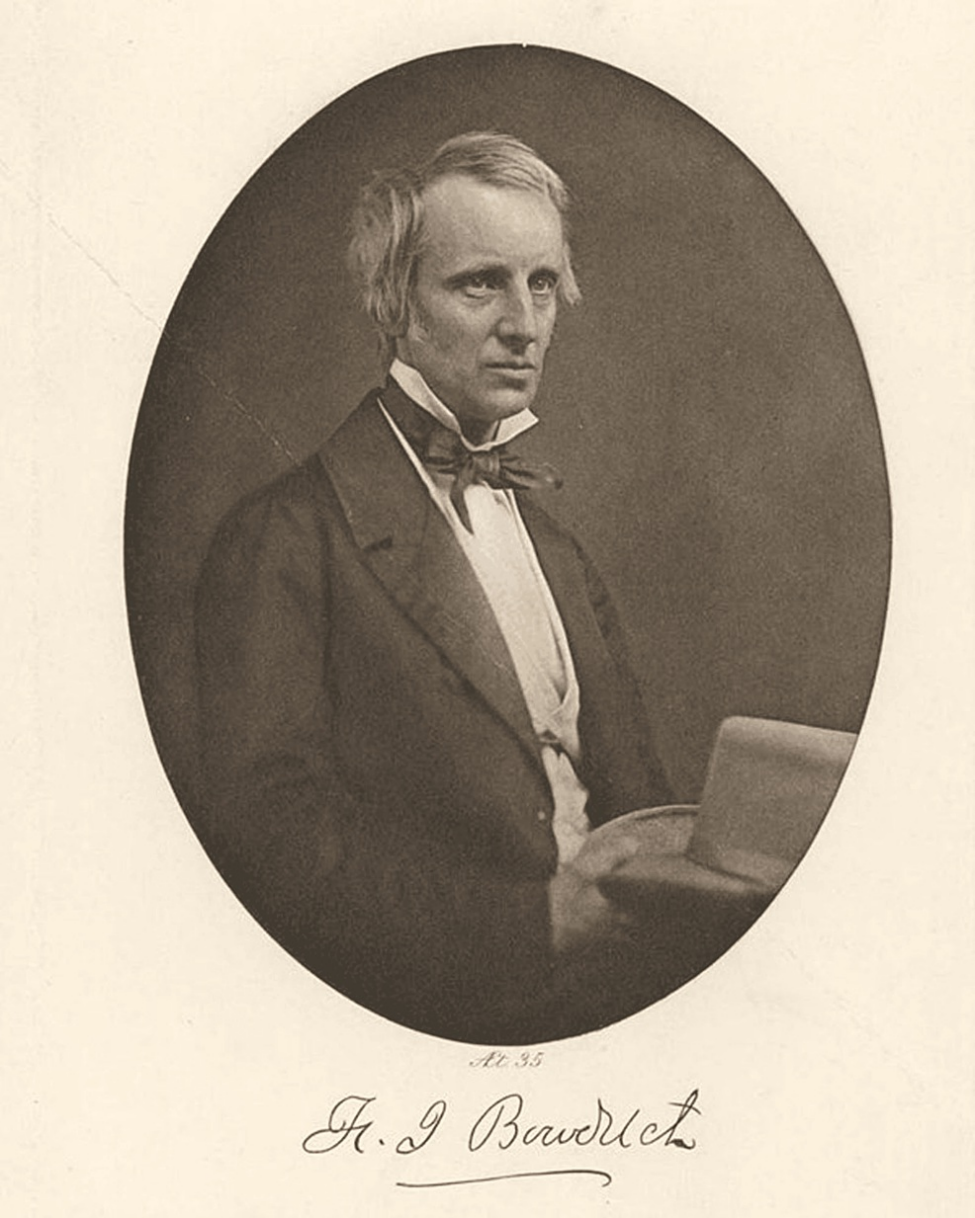
Henry Ingersoll Bowditch’s (1808-1882) portrait by the Massachusetts Historical Society.

The American mathematician Nathaniel Bowditch (1773-1838) is credited as the founder of modern maritime navigation after his book “The New American Practical Navigator,” which was published in 1802. Nathaniel replaced the “dead reckoning” method with a lunar method to find longitudinal orientation more reliably, gaining global fame [1]. Nathaniel Bowditch was the fourth of seven children in his family, born in Salem, Massachusetts. His family was large too, and Henry Ingersoll Bowditch (1808-1882) was the third among Nathaniel’s six sons and two daughters. Although the young Nathaniel had worked hard in his father’s cooperage and as a bookkeeper, Henry studied at the Boston Latin School and then at Harvard College in 1828 [2]. Life in the United States of America demanded activity in the scientific and professional life of physicians. A great number of them travelled to Europe for additional medical education. Polished by their European education, American physicians excelled and were placed in the race for leadership in medicine and surgery [3]. Henry Ingersoll Bowditch was such a man (Figure 1).

## Review

During 1828, Henry had decided to enter Harvard Medical School, most probably being influenced by the influential, reformer, and highly respected physician, professor of clinical medicine, James Jackson (1777-1867), to whom Henry attributed a book with his notes titled “Note Book on Lectures of James Jackson” [4]. For a two-year period, Henry moved to Paris to study under French physician, clinician, and pathologist Pierre Charles Alexandre Louis (1787-1872), known for his studies on tuberculosis, typhoid fever, and pneumonia and as the founder of the “numerical method.” Henry became a member of the “Société Médicale d’Observation” (Society for Medical Observation) and adopted Louis’ methodology on patient observation, correlations of symptoms with lesions discovered at autopsy, epidemiology, and the application of statistical methods to treatment decisions. After returning to Boston, Bowditch was appointed as an admitting physician at the Massachusetts General Hospital [2,4]. Harvard was the scientific epicenter for Bowditch’s family. In 1806, Nathaniel declined the position as Harvard chair of physics and mathematics, Henry became a professor of medicine at Harvard Medical School in the 1840s, and Henry Pickering (1840-1911, Nathaniel’s grandson and Bowditch’s nephew) was the founder of the first physiology department at Harvard [2].

Henry had encountered stethoscope during his French education. Invented by French physician and musician René-Théophile-Hyacinthe Laennec (1781-1826) and described in his 1819 book on auscultation, the stethoscope was destined to become one of the most employed tools of a physician during patients’ examination. Henry’s experiences in several French hospitals provided him with an excellent opportunity to learn how to use this new diagnostic tool, making him the first American physician to become proficient in its use. He eventually wrote a treatise, titled “The Young Stethoscopist,” in 1846 to encourage American physicians and students to use the stethoscope during their examination when searching for symptoms that might be caused by disorders of the heart or lungs. This is how the use of stethoscopes started across the United States of America [2,4-5]. To learn how to understand sounds during auscultation by a book was rather dubious, and physicians were in need of vast practice. A stethoscope provided a method of communication between acoustic symptoms and related diagnoses. The murmurs of the heart and the new tool somehow stimulated an interest in valvular heart disease, which was a significant clinical problem of the era. According to contemporary views, the mere presence of a heart murmur was considered a strong indicator of a serious heart disease. A stethoscope, though, proved that some kind of murmurs did not imply a firm poor prognosis [4].

**Figure 2:**
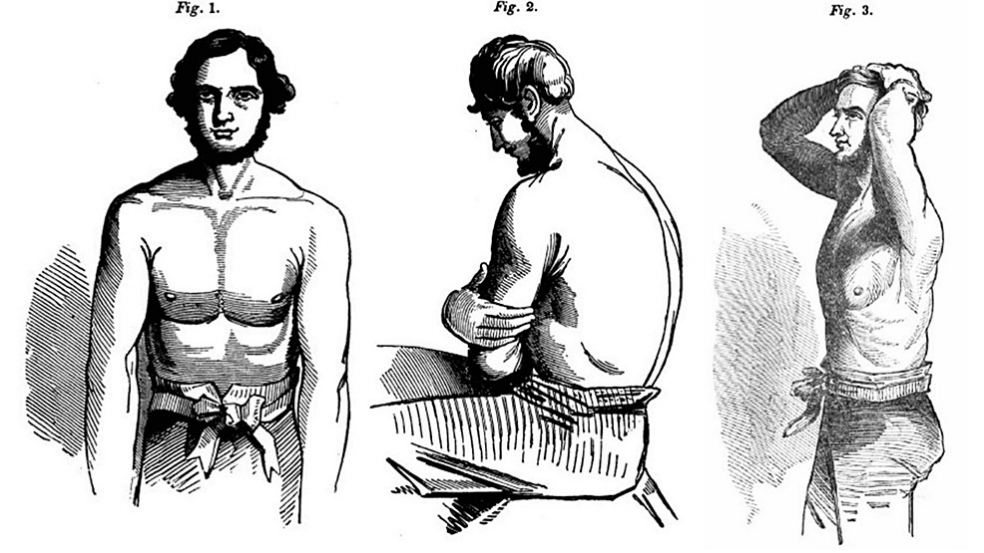
Positions of the patient to provide a smooth auscultation procedure, as they were presented in the treatise “The Young Stethoscopist” (1846). Note: Image sourced from “The young stethoscopist, or the student's aid to auscultation,” by Bowditch HI, 1846, J. & H.G. Langley, New York, NY [5]

**Figure 3:**
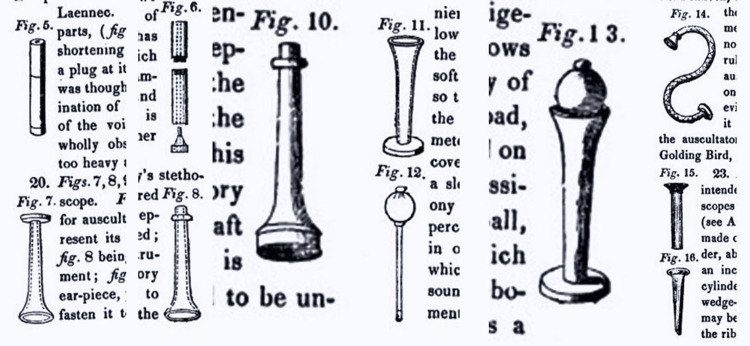
Various types of stethoscopes being in use during the 19th century, as they were presented inside the treatise “The Young Stethoscopist” (1846). Note: Image sourced from “The young stethoscopist, or the student's aid to auscultation,” by Bowditch HI, 1846, J. & H.G. Langley, New York, NY [5]

Henry Bowditch considered auscultation as an art as he had stated in the preface of his book. He suggested that the patients be uncovered or clothed with a thin, single dress, in a position to allow easy auscultation. To emphasize both nakedness and position, he presented a simple figure of a man (Figure 2). Inspection for symmetry and diseases was also proposed. Palpation to feel lung diseases and mensuration of the chest could also reveal some issues. For the main examination, although the author supported the stethoscope, he noted that immediate auscultation directly to the chest, using the physicians’ ear, was also an acceptable method. To help young colleagues to get acquainted with the stethoscope, he presented inside thedemonstrated various types being in use (Figure 3). Bowditch proposed that respiratory murmur is a delicate, breezy sound, influenced by age, position of auscultation, disease (pneumonia, pleurisy, pneumothorax, and tumor), emotions, rapidity of breathing, actions of the heart, and injury (muscle or bony). He thoroughly presented local anatomy such as aorta and thorax segmentation to better explain the sound heard. A series of different types of sound were marked, such as bronchophony, egophony, pectoriloquy, rales of bronchi, metallic echo, mucous rale, and gargling. He had presented various diseases and how they should be heard through a stethoscope, such as pneumonia, pleurisy, pericarditis, phthisis, endocarditis, hypertrophy of the heart, valvular diseases, polypi of the heart, and aneurisms of the aorta, stating their physical signs too. Various chapters such as obstetrics and veterinary auscultation enhanced knowledge for younger physicians.

On 22 February 1854, Henry read before the Boston Society for Medical Improvement “Comments on a Hitchcock Paracentesis Thoracis: Four Times Performed on the Same Person.” He used thoracentesis as a treatment for pleural effusion, believing that this method with the use of a trocar and a suction pump was better than a method with the scalp. A small trocar could be easily introduced, with almost no wound and less possibility of a fistulous opening remaining and no air to be inserted into the pleura. Henry noted that he had “never seen any evil result” [6]. His papers on tuberculosis were also among his most important essays. The last paper he had publicly announced was the “Open Air Travel as a Means on Cure for Consumption” as a method to confront hemoptysis and the symptoms of tuberculosis, as his father did and successfully arrested his disease [7].

After the Emancipation and the end of the American Civil War, Henry tried to reorganize medicine and improve relations between physicians in the North and South, while simultaneously, he proposed a code of medical ethics. Henry was among the founders of the Massachusetts Board of Health, in which he served as its first president in an effort to promote public health through personal sanitation and city hygiene. He noted that medicine and politics combined should promote sanitary laws even though they may cause conflicts in the financial and property rights of the citizens [1,7-9].

Discussion

Henry Ingersoll Bowditch presented after the suggestion of the School Committee of Boston a sketch of the life of his father Nathaniel, a practice that seems to be a tradition among the members of the Bowditch family [10]. For Henry’s life, there exists another treatise named “Life and Correspondence of Henry Ingersoll Bowditch” written by Vincent Yardley Bowditch in 1902 [11]. Henry’s practice with the stethoscope was also highlighted within his book “The Young Stethoscopist or the Student’s Aid to Auscultation,” aiming to promote further stethoscope use in the United States of America [12]. When he was writing about sanitary issues, he was always noting that his address was not only to local authorities and physicians but also to activate all people involved, citizens, physicians, state employees, and politicians, toward significant hygiene problems [13].

His practice in thoracic diseases was due to an unlucky incident, when during a midwifery operation in 1852, an injury caused a septic infection resulting in a finger loss [14]. On at least five treatises, Henry discusses his experience on pleuritic effusions and the necessity of their removal. Since the days of the majestic ancient Greek physician Hippocrates, thoracentesis had been performed already, a procedure known to all European physicians. It was the stethoscope that made clear its diagnosis, and Henry Ingersoll Bowditch used both the new tool and the ancient procedure [14,15]. The American surgeon Paul Fitzsimmons Eve (1806-1877), who had practiced battle surgery during the Polish-Russian War, had emphasized the significance of thorax examination and celebrated paracentesis thoracis. The importance of this method during the American Civil War was that it was being noted as among the life-saving procedures performed by military surgeons [3].

Henry had an ideal childhood and a luxurious, wealthy late life, enabling him to have a memorable education and a profound commitment toward public health reforms. It seems that he was a humanist, fond of medicine, and interested more in solving problems, rather than in practicing surgery due to his repugnance for scalpel use (Table 1) [14].

**Table 1:** Chronological overview of Henry I Bowditch’s life accomplishments and events. Source: [1]

Henry I Bowditch’s life accomplishments and events
8 August 1808: Born to Nathaniel and Mary Ingersoll Bowditch in Salem, Massachusetts, United States of America
1828-1832: Enters Harvard Medical School studying under James Jackson
June 1832-1834: Travels to Paris with Oliver Wendell Holmes to study at Ecole de Médecine with Pierre Charles Alexandre Louis, renowned physician and promulgator of the “numerical method”
Spring 1834: While travelling in Italy, he hears of his mother’s death from consumption
1835-1838: Appointed admitting physician at Massachusetts General Hospital (MGH), joins Massachusetts Medical Society (MMS), co-founds the Society for Medical Observation, and proposes useful changes to the laws regarding smallpox vaccination
1841-1849: Working through the MMS, promotes laws to make Massachusetts the first state to record public vital records including births, marriages, and deaths; petitions state legislature to create a Sanitary Commission for the sake of public health; resigns from MGH because of exclusion of Black patients; actively supports the abolition movement and coordinates movements of the “Underground Railroad”; publishes The Young Stethoscopist; and continues to translate Dr. PCA Louis’ work from French to English
1849-1854: Composes the Bowditch Book as secretary for MMS, compiling three volumes of important medical papers, patient case records, and other irreplaceable documents; founds the “Boston Anti-Man-Hunting League”; and participates in abolitionist demonstrations
1854-1862: Focuses his study on consumption, its prevalence, and communicative potential in relation to environmental conditions; delivers findings to the MMS in “Topographical Distribution and Local Origin of Consumption in Massachusetts”
1862-1869: Advocates for an “Ambulance Bill” to improve wartime medical care and sanitary conditions; appointed to the first chair of the Massachusetts Board of Health
1870-1881: Helps to maintain safe storage of vital MMS documents and research reports in addition to founding the Boston Medical Library Society; appointed by President Hayes to be a member of the new National Board of Health; acts to promote the inclusion of females in medical education and physician practice
14 January 1882: After a fall on an icy street in 1879, Bowditch’s health steadily declines, and he died in Boston in 1882

## Conclusions

Henry Ingersoll Bowditch’s opinions concerning public health were not in vogue during his time. However, alongside his moral brilliance, they served as examples for the future and advanced social medicine. The introduction in the United States of America of thoracic examination with the use of a stethoscope and of thoracic paracentesis developed from ancient times to its accurate modern form by Bowditch allowed him to be considered as one of the most important figures in general medicine, while he must be credited as a pioneer of the use of stethoscope and auscultation in the United States of America.
